# Healthcare professionals’ perception of barriers and facilitators for care coordination of older adults with complex care needs being discharged from hospital: A qualitative comparative study of two Nordic capitals

**DOI:** 10.1186/s12877-023-03754-z

**Published:** 2023-01-19

**Authors:** Janne Agerholm, Natasja Koitzsch Jensen, Ann Liljas

**Affiliations:** 1 Aging Research Center, Karolinska Institutet, Stockholm, Sweden; 2 Department of Global Public Health, Karolinska Institutet, Stockholm, Sweden; 3 Sector of Social Medicin, Copenhagen University, Copenhagen, Denmark

**Keywords:** Care coordination, Denmark, Frail elderly, Home care services, Hospitals, Nursing staff, Patient discharge, Qualitative research, Sweden, Transitional care

## Abstract

**Background:**

The handover of older adults with complex health and social care from hospital admissions to homebased healthcare requires coordination between multiple care providers. Providing insight to the care coordination from healthcare professionals’ views is crucial to show what efforts are needed to manage patient handovers from hospitals to home care, and to identify strengths and weaknesses of the care systems in which they operate.

**Objective:**

This is a comparative study aiming to examine healthcare professionals’ perceptions on barriers and facilitators for care coordination for older patients with complex health and social care needs being discharged from hospital in two capital cities Copenhagen (DK) and Stockholm (SE).

**Method:**

Semi-structured interviews were conducted with 25 nurses and 2 assistant nurses involved in the coordination of the discharge process at hospitals or in the home healthcare services (Copenhagen *n* = 11, Stockholm *n* = 16). The interview guide included questions on the participants’ contributions, responsibilities, and influence on decisions during the discharge process. They were also asked about collaboration and interaction with other professionals involved in the process. The data was analysed using thematic analysis.

**Results:**

Main themes were communication ways, organisational structures, and supplementary work by staff. We found that there were differences in the organisational structure of the two care systems in relation to integration between different actors and differences in accessibility to patient information, which influenced the coordination. Municipal discharge coordinators visiting patients at the hospital before discharge and the follow-home nurse were seen as facilitators in Copenhagen. In Stockholm the shared information system with access to patient records were lifted as a facilitator for coordination. Difficulties accessing collaborators were experienced in both settings. We also found that participants in both settings to a high degree engage in work tasks outside of their responsibilities to ensure patient safety.

**Conclusions:**

There are lessons to be learned from both care systems. The written e-communication between hospitals and home health care runs more smoothly in Stockholm, whereas it is perceived as a one-way communication in Copenhagen. In Copenhagen there are more sector-overlapping work which might secure a safer transition from hospital to home. Participants in both settings initiated own actions to weigh out imperfections of the system.

**Supplementary Information:**

The online version contains supplementary material available at 10.1186/s12877-023-03754-z.

## Background

The hospital discharge process and subsequent transfer to home care for older people with complex care needs (both medical and social care needs), involves multiple activities which requires extensive coordination between several care teams [1, 2]. Linking planning and management of the activities involved has the potential to generate a coherent scheme of management, which can positively influence management continuity if conducted well [3]. Previous research has demonstrated that clear communication between the involved care teams is essential [4–7]. The discharge planning should further start as soon as the older person has been admitted to hospital so that necessary services can be identified [4].

Poor hospital discharge planning tends to cause delays in communication between hospital-based healthcare professionals and health and social care providers in the community, negatively affecting the management of the patients [8–12]. Subsequently, hospital readmission is common [13, 14]. However, there is evidence suggesting that post-discharge adverse events could have been prevented through comprehensive discharge planning [15]. Carefully planned and performed discharges have also the potential to improve both patients’ quality of life [16] and satisfaction with the process [17]. Providing a coordinator designated to manage the hospital discharge planning has shown to facilitate the process, however, this is often not standard [18, 19].

Studies have shown that nurses are often exposed to heavy workload with little or no time to prepare patients and their family members for the hospital discharge, causing unnecessary stress amongst patients and their close relatives [4, 20]. The hospital discharge process can be especially challenging for family members of older adults with cognitive decline who previously have been reported to have many unmet needs that require individual assistance in the immediate time post discharge [21].

Many qualitative interview studies on hospital discharge provide the views of the patient [22–26], and while the patient’s view and patient involvement is very important for a safe transfer [5, 27], we have found less research investigating the process from healthcare professionals’ perspective. Especially research combining the views from both the hospital workers and the home healthcare workers. Providing insight to the care coordination during the discharge process from healthcare professionals’ views on both sides is crucial to show what efforts are needed to manage the handover from hospital to home based healthcare, and to identify strengths and weaknesses of the care systems in which it is provided. This is important as failure in the coordination can negatively affect the patient [8, 9]. The purpose of this study was to investigate two different ways of organising the discharge process and the coordination related to a handover from hospital to homebased care to see if we can find common themes for what facilitate or compromise a safe return for the patients.

In this study we focus on the care coordination between nurses (and in Sweden also two assistant nurses working as care coordinators) in hospitals and nurses in home healthcare. The nursing staff in the hospitals are the ones responsible for the discharge process at the hospitals, as well as the ones handing over information to municipal and primary care. The nurses in the home healthcare settings are the ones responsible for following up the care needs after discharge. The coordination and communication between these two groups are therefore important to achieve a safe return from hospital to home for the patient.

A comparison of the views of healthcare professionals working in different care systems has the potential to bring further clarity to and suggestions on how coordination in each of the systems can be further developed and improved as well as identifying context and non-context dependent variables.

### Differences and similarities in the organisation of health and social care in Copenhagen and Stockholm

There are many similarities in the general organisation of health and social care in Denmark and Sweden. In both countries, health and social care is universal, tax-funded, and de-centralised where the regions in general are responsible of healthcare and the municipalities of social care. However, there are also differences. In Copenhagen, and in Denmark in general, the home healthcare is provided by the municipality and is organised very much like the home help services. There is a municipality-based need assessor (a nurse) in charge of both assessing the patient’s needs and coordinating the services provided by the home care nurses.

In Stockholm, the Region is responsible for home healthcare and the primary care clinics oversees home healthcare services. Each patient can choose a permanent care-contact [fast vårdkontakt] to coordinate different healthcare appointments. A permanent care-contact can also be appointed if necessary. In most cases the permanent care contact is a district nurse at the primary care clinic where the patient is listed. The district nurses at the primary care clinics are also the ones responsible for providing home healthcare to the patients.

### Discharge of older patients with extensive care needs in Copenhagen:

In Copenhagen, the regions and the municipalities are obligated to collaborate on developing mandatory healthcare agreements that sets the framework for how the coordination on treatment, prevention, discharge and rehabilitation is to take place. Most of the information between hospitals, municipalities and primary care physicians is disseminated through a shared IT-system called MEDCOM.

At the time of admission to the hospital an automatic message is sent to the patient’s home municipality with information about the date of admission. If the hospital anticipate that the patient will leave the hospital with changed functional level, they are obliged to send a care plan (Plejeforløbsplan, PFP by Danish acronym) to the municipality as soon as possible and no later than 48 h after admission [28]. The care plan contains information on the patient’s functional level, the care, medication, and home help services that the hospital’s healthcare professionals recommend after discharge. An assessor at the municipality receives the care plan and start planning for the patient’s return to home. On the day of the discharge, a discharge report, made by the hospital nurses, is sent to the municipality. This information is automatically sent to the municipality and activates the municipal services.

### Discharge of older patients with extensive care needs in Stockholm:

In Stockholm, a new law on hospital discharge was introduced in 2018. This involves hospital healthcare professionals to provide an estimated discharge date within 24 h after a person has been admitted to the hospital. If the patient is likely to need healthcare after the hospital stay, information about the patient's health status is shared with their primary care clinic, which is responsible for the care post hospital discharge.

The primary care clinic will assign a home healthcare nurse to continue providing care in the patient’s home. If the patient will require post hospital medical care that is either considerably greater than prior to the hospital stay, or the person has not received home healthcare in the past, a care planning meeting will be arranged by the home healthcare nurse in the patient’s home upon their return. The home healthcare nurse liaises with the social care manager who attends the meeting if social care seems to be needed. If the older person will not need any home healthcare but will need social care to manage everyday life following the hospital stay, healthcare professionals at the hospital may liaise directly with the social care manager and arrange for such meeting at the hospital prior to discharge.

## Methods

### Aim

The aim of this study is to examine and compare the healthcare professionals’ involved in the coordination perceptions on barriers and facilitators of care coordination for older patients with complex health and social care needs being discharged from hospital in two different healthcare settings: Copenhagen (Denmark, DK) and Stockholm (Sweden, SE).

### Design and data collection

This is a qualitative interview study based on individual interviews. Recruitment and data collection took place in the last part of 2018 (October-December) in Copenhagen and early 2019 (January-April) in Stockholm. The delay in data collection in Stockholm was to ensure that participants had some experience of and were able to report from the perspective of the new hospital discharge routine implemented in autumn 2018. Twenty-seven healthcare professionals (12 hospital nurses, 2 hospital assistant nurses and 13 home healthcare nurses) involved in the coordination of the discharge process were interviewed (Copenhagen *n* = 11, Stockholm *n* = 16). Purposive sampling using a snowball approach was used and continued until saturation was reached[29]. Study participants were firstly identified through the researchers’ network and variation in relation to different organisations and different job positions within the organisations were prioritised.

A semi-structured interview guide was developed based on previous research to address the research questions [1, 8, 30, 31]. The interview guideline included questions on the health professionals’ contributions, responsibilities, and influence on the discharge process. They were also asked about collaboration and interaction with other professionals involved in the process (interview guide can be found in supplementary file). The interview guide was pilot tested in both Denmark and Sweden.

The interviews were undertaken 1-to-1 and face-to-face in the local language by a skilled qualitative researcher in each city (AL (PhD), NKJ (PhD)). All interviews were audio-recorded, field notes were taken after each interview and the interviews were transcribed verbatim. The interviews were anonymised and the participants given id-codes. The interviewers and (JA) reviewed transcripts for accuracy. Further information about the researchers’ background and preconceptions can be found in the supplementary file.

### Analyses

Interview data were analysed using a thematic analysis that entails six different phases: familiarising oneself with the data, generating initial codes, searching for themes, reviewing the themes, defining and naming the themes and lastly reporting of the findings [32]. The transcripts in Danish were read by NKJ (PhD) and the Swedish transcripts were read by AL (PhD). All transcripts were read by the bilingual Danish-Swedish researcher JA (PhD). Themes and sub-themes that emerged were discussed within the team. To ensure credibility [33] two interviews from each country were coded by two researchers separately (investigator triangulation), to compare codes and sub-themes. After agreement, AL then coded the Swedish interview and JA coded the Danish interviews. Participants who agreed to be contacted and were accessible were provided the opportunity to comment on codes and results before drafting the manuscript (member checking).

### Description of the participants from Copenhagen

In Copenhagen, 5 of the interviewed nurses worked at hospitals and 6 worked in the municipality (either in the central administration or in home nursing care centres).

Two of the nurses at the hospital were coordination consultants working across clinics assisting the clinical based nurses with the discharge process when needed. One of the hospital-based participants was a follow-home nurse whose work was to coordinate the discharge and follow the patient home, if needed. This service is available on hospitals in the capital region since 2009 and are available for the frailest older people to facilitate a safe transition from the hospital to the home. The remaining two nurses were based at two medical clinics and involved in the discharge of patients at those clinics.

In the municipality, two of the interviewed nurses were assessors, responsible for the initial assessment of home care. Two participants were discharge coordinators. The last two municipality-based participants were home care nurses who meet patients in their homes following a discharge.

### Description of the participants from Stockholm

Of the 16 participants in Stockholm, 9 worked in geriatric clinics at hospitals and 7 were based at primary care clinics. All participants in primary care were home healthcare nurses whose daily work involves logging on to the electronic system shared with healthcare professionals at the hospitals for updates on planned hospital discharges, and to visit and provide medical care to patients in their homes. Among the 9 hospital-based participants, 6 were nurses at geriatric clinics with care coordination responsibilities, and 3 participants worked exclusively as care coordinators of which one had a background as a nurse and two had previously worked as assistant nurses.

## Results

Three main themes related to facilitators and barriers for coordination during discharge, emerged from the data analyses: intended communication ways, organisational structure and supplementary work. Themes and subthemes can be seen in Table 1.

**Table 1:** Description of the themes and sub-themes that emerged from the data analyses

	Themes	Sub-themes	Description
Facilitators and barriers	Intended communication ways	Primary communication	Description of the primary way of communication
		Coordination meetings across organisations	Meetings related to general communication and collaboration (not related to specific patients). This was only found in Sweden
		Insufficient information/communication regarding the patients care needs after discharge	When there is lacking information in the communication between the different actors or when information is sent out to late
		Accessibility of collaborators	Possibility to reach other actors/collaborators when needed
Facilitators and barriers	Organisational structure	Guidelines and regulations	The guidelines and regulations provided
		Extra resources for strengthening the coordination	Specific projects/extra resources put in place to facilitate better coordination
		Uncertain responsibility	Uncertainty about who is responsible in specific situations
		Staff influence on coordination and information	The staff’s possibility to influence the coordination and the information provided
Facilitators	Supplementary work	System knowledge	Knowledge about the system and the working conditions/responsibility of other actors
		Additional communication initiated by staff (Not requested or part of general guidelines)	In cases where primary communication is not sufficient staff initiate communication by their own to get the information needed or to secure that important information is passed on
		Work beyond duty	When the staff initiate responsibility on their own. Take responsibility in situation when it becomes unclear who is responsible or in situations where the system does not work. Efforts put in place by the personnel to balance system errors or to help smooth the coordination in addition to the official guidelines or communication ways

### Intended communication ways

Intended communication ways refers to the primary ways of communicating between the different participants involved in the discharge process of older adults. It also includes aspects related to lack or delay of communication and dissemination of information between actors. The sub-categories can be found in Table 1.

### Primary communication

In both Copenhagen and Stockholm, the participants reported that communication between hospitals, municipality and primary care physicians/nurses was mainly handled through shared electronic systems. In Copenhagen, the system automatically sends out relevant patient-related information to the municipality and the primary care physician where the patient is admitted. However, the full patient record is not shared. In Stockholm, much more information is shared between hospitals and primary care clinics and the primary care clinic often have access to the full patient records. The participants from both Copenhagen and Stockholm generally found their respective system to be very useful tools.

The hospital nurses in Copenhagen experienced the electronic communication very much as a one-way communication and appreciated the more face-to-face collaboration with discharge coordinators from those municipalities that regularly visited the hospitals.

*“There is one municipality that comes out every Tuesday and Thursday, an assessor who attends board meetings. Then they come out and talk to the patients who are from that municipality. There we also have a partner we can confer with. How do they know the citizen from the municipality and how do we see the citizen here? It's a really good sparring partner. For example, I have to discharge a patient now, where I have sent a PFP [care plan] to the municipality, but it is very much a one-way communication. I must assume that, when I have sent that message, the municipality has put on the extra help needed. I feel it is very insecure, because I have no experience from the municipality, so I have not seen what they do and what they do not do. I just have to trust the PFP I sent away, that they do what I have said. And I find that very fragile.”*

*(Hospital nurse DK, int5).*

In Stockholm, both healthcare professionals at hospitals and in primary care have access to the same system called Web-Care. Web-Care contains up-to-date information about the patient’s diagnoses prescribed medicine and patient records from the hospitals. In addition to having access to shared medical information about the patient, all participants reported that they use the system to communicate via a chat function. They expressed that they liked the possibility to communicate through this function, however, some nurses in primary care found it hard to find time to check and respond to the communication from the hospitals. Likewise, some participants based in hospitals found it stressful that they did not receive responses from primary care nurses confirming that they received the message(s) sent or confirming that they will provide the suggested home healthcare to a patient on discharge.

*“We have to log in to Web Care at least twice a day. I try because I know I have to, but it is not easy, it is not easy because sometimes you have ten patients [in WebCare] that you have to open and it's not just to open. When I press and open, then I have to continue, I cannot leave. Sometimes I have someone coming in or a patient comes [to the health center], then you have a patient waiting out there, and then I leave the case. On the other hand, the hospital or geriatrics, they can see that I have been inside the patient's case, but I did not have time to answer, and then they get a little annoyed. They see that I have been inside but not answered. I think it's a bit [stressful] not to have time to answer everything in Web Care.”*

*(Primary care nurse SE, int1502).*

### Cross-sector coordination meetings

In both Copenhagen and Stockholm participants reported on meetings across organisations where more general questions regarding the coordination between actors such as the municipalities and hospitals are discussed. These meetings were seen as facilitators for better communication and collaboration between the different agents.

Apart from the cross-sector coordination meetings, participants in Stockholm also reported participating in informative sessions about the newly implemented law on coordination [LUS] where they obtained a better understanding of each other’s roles.

### Insufficient information/communication regarding the patients care needs after discharge

Almost all participants in Copenhagen mention the shared electronic information system as something that facilitate smooth transfer of information, however, many of the participants from the municipalities in Copenhagen reported that they sometimes lacked information in the care plans. In these cases, the visitation nurses in the municipality had to call the hospital by phone to get the additional information. In most cases the participants said that they received the information needed when calling the discharging nurse.

The lack of information was an even greater problem for the home nurses, sometimes were send out to patients with very little information before the first meeting.

*“There is a bit ‘tabula rasa’ about it when you get out. It's like a wiped-clean blackboard. We have almost no idea about what is wrong. I often see myself as a detective trying to figure out what's really wrong here.”*

*(Municipal nurse DK, int2).*

The home nurses further explained that the discharge report contains some information about the reason for admission, however, they seldom take up other diagnoses, not related to the admission, that the patient might have.

### Accessibility of collaborators

Although most of the communication between the actors in Copenhagen is handled electronically through the shared it-system, the municipality sometimes needs to confirm information or ask for further information from the hospitals. In those cases, accessibility to the discharging nurse becomes important for the staff in the municipality.

*“Sometimes you get through easily, other times you can spend an hour getting in contact with the right person on the hospital. It is a barrier, that I clearly experience. The contact is not straightforward. It may not be the nurse who knows the patient you get hold of on the phone, but another one, that have to read through the medical record again to see what has happened.”*

*(Municipal nurse DK, int1).*

The primary care physician is the one responsible for the medical aspects of the care plan after discharge. The municipality does not have access to the discharge summary, also called epicrisis, sent to the primary physician and some of the participants mentioned that they sometimes lacked information regarding for example discontinuation of medicine in the care plan. They see that a change has been made in the shared medicine card, but not why or when. In cases where they are uncertain about for example the data in the shared medicine card they need to contact the primary care physician. In all the interviews where the participants sometimes had to contact primary care physicians, they mention difficulties with accessibility.

*I do not think they are super accessible; I have to be honest. Talking to a physician is not easy. Then I call a secretary, who does not even have a health education necessarily, and then "Yes, but then you have to call during the physician's phone hours". Well, it cannot wait until the doctor's phone hours. "Well, we have closed the phones now". But it is 12 o’clock!? I cannot call 1813 [for evening and weekend emergencies], there are no physicians either, so what do you want me to do? Some physicians also just shut down their phone….They are difficult to get hold of. So, it is difficult to get a medical assessment when we are out there with a patient in poor health.*

*(Municipal nurse DK, int2).*

Similarly, the hospital nurses mention that the primary care physicians do not always follow up on medical actions asked for by the hospital physicians. For example, if further blood samples are needed after discharge or if they need to call in the patient for a follow up blood pressure test. Their experience is that the primary care physician waits for the patient to contact them, before reacting to the suggestions in the epicrisis.

In Stockholm, many participants expressed frustration not getting hold of cooperating actors based elsewhere. This was particularly reported by the nursing staff at hospitals who wanted to get in touch with primary care nurses:

*So, it’s like the city districts, they are very different. Some do not even sign. It is like they do not care at all, because they want to show that this does not work out, maybe, I do not know. But it’s like a chase for me. It’s their patients and you have to chase around, and you never get hold of them and there are no [telephone]numbers. You write in Web Care and such, but you do not know if they have seen it or so.*

*(Hospital nurse SE, int2304).*

Some participants in Stockholm also reported on having difficulties getting hold of social care managers, physiotherapists etc. who do not automatically have access to the shared electronic system yet often need to be contacted for the arrangement of patient care planning meetings.

In Stockholm, it was reported that nursing staff at hospitals sometimes faced extreme challenges getting access to and collaborating with patients’ primary care clinics. Nursing staff from the Stockholm inner city gave examples of older adults, with comprehensive home healthcare needs, who were prompted to become patients at different primary care clinics:

*We had a patient, who had had the same primary care clinic for 35 years and got a probe, a PEG [Percutaneous Endoscopic Gastrostomy] and an ostomy bag because he had had cancer, and then the primary care clinic thought he should be re-listed somewhere else, even though he had been listed at the same primary care clinic for 35 years and had a doctor there and everything; yet the primary care clinic wanted to pass him on to another clinic, That does not feel really.., it will not be a safe discharge because then the patient gets a new doctor, gets new district nurses and a completely new primary care clinic that he does not recognize.*

*(Hospital nurse SE, int2803).*

### Organisational structure

Organisational structure refers to how the responsibility is divided between actors as well as what regulations and guidelines are in place to help the participants manoeuvre in the system. It involves resources put in place to help facilitate care coordination as well as accessibility of these resources. The theme also covers aspects related to when regulation or guidelines are missing or when it becomes unclear who is responsible for what actions in the discharge process. The sub-categories can be found in Table 1.

### Guidelines and regulations

In both Denmark and Sweden there are regulations regarding the discharge process. At the time of the interviews, the new Swedish law on coordination at discharge had recently been introduced, causing some confusion and misunderstandings according to a few of the participants.

Some participants in Stockholm further mentioned that occasionally some municipalities seem to misuse the system:

*“Sometimes it can feel like some municipalities may take advantage of that, now it is five days until they become liable for payment, then they wait the five days to take the patient home.*

*(Hospital nurse SE, int1017).*

In Copenhagen, the coordinating consultant could be consulted if there were any disputes between the hospital and the municipality regarding responsibilities or time limits. The coordination consultant was seen as a good help for the discharging nurse and were seen as very knowledgeable on the rules and regulations.

### Extra resources for strengthening the coordination during discharge

The hospital nurses in Copenhagen mentioned that some municipalities had discharge coordinators coming to the hospital on a regular basis (2 times a week) visiting the patients from that municipality. They felt that these regular visits to the clinic facilitated more and better communication between the nurses at the hospital and the municipality. In both cases these extra resources were seen as positive for the patients and as something that increased collaboration and communication between the different actors. It made the collaboration run more smoothly and gave the possibility for a feedback-loop to the hospital, something that the nurses felt were missing through the electronic system.

*I think it's nice that one municipality is coming out [to the hospital]. I think it really gives us some peace too. ……..It is nice when they come. Then the assessor [discharge consultant from the municipality] goes out and talks to the patient, and then a relative can take part. Then they can do it. Then the assessor comes back and tell me what the plan is, so that I can type it into the system.*

*(Hospital nurse DK, Int5).*

Other participants mentioned previous resources that had been part of the system but was no longer available. In general, most of the participants from Copenhagen mentioned different smaller projects that seems to have been introduced for a limited time to improve the care coordination for older people.

Also in Stockholm, there were examples of differences in resources between municipalities. The participants in Stockholm mentioned two initiatives regarding safe transition between hospital and home. One that was implemented in some of the city districts of Stockholm municipality and one in a municipality in the southern part of Region Stockholm.

### Unclear responsibility

Unclarity on responsibilities was mentioned more frequently by the respondents from Stockholm, often related to the newly changed legislation, however, it also came up in some of the interviews from Copenhagen.

*I would like more information from the hospital in general. Most often medicine is discontinued and then some medical treatments are stopped at the hospital without us knowing why. The citizen does not know either. The patient’s primary care physician also cannot find the epicrisis and will not take responsibility and then I have to call the hospital again, and I actually think I experience this relatively often. Where suddenly it is the home nurse who has to be the person in charge of figuring out what the right treatment is, even though it is actually the physician who is responsible for treatment.*

*(Municipal nurse DK, int1).*

In Stockholm there were mainly two areas where there was uncertainty about responsibility. The respondents in primary care reported that there were areas where they did not know if the responsibility was the provider of home healthcare or the provider of home help services. Another area was related to the responsibility of the primary care nurse being the coordinator of the discharge process and transition from hospital to home. This responsibility was placed on primary care with the new law (LUS).

### Supplementary work

Aspects related to the supplementary actions covers aspects like staff’s knowledge about the system and the different actors in the system. It also includes actions and responsibilities taken by the staff that lies beyond the regulations and guidelines to secure a safe discharge or actions that is felt needed because of system failures. The sub-categories can be found in Table 1.

### System knowledge (Understanding of other care staff’s work duties)

An aspect perceived as facilitating good coordination among the participants in Copenhagen was the staff’s knowledge about the work of other actors in the system. From the hospital-based nurses’ perspective it was important to know that the municipalities do not have access to the patient hospital records and are therefore dependent on the information provided in the care plan and the discharge report. This was reported to motivate them to provide more in-depth descriptions in the care plan. They also considered it important to know about what type of social services the municipalities provide to understand what type of help they can request in the care plan and what the patient can expect upon discharge. Different types of more advanced care sometimes needed to be introduced to the municipal nurses at the hospital if they were not used to those procedures. Information about the type of healthcare services usually provided helped the hospital nurses to identify when an introduction to a new care regimen was needed.

The municipal nurses expressed that knowledge about how a hospital works and what type of services is provided at the hospital helped them understand what the hospital can do for the patients and what they do not do for the patient. They had a better understanding for when information might be missing, and what type of information could be missing.

### Additional communication initiated by staff

Some respondents in Stockholm reported establishing contact with the home help teams that provide social services if they happen to be at the patient’s home at the same time, but they have no formal collaboration:

*What you cannot count on, is that home care, for example, is in place and meet [the patient]. And you can understand that because they have a lot of things to do and they may be delayed at a patient and then everything falls. So that, you cannot count on, but otherwise I feel that if they say they will follow it up, then I think they will.*

*(Hospital nurse SE, int1017).*

Several of the nurses at the hospitals in Copenhagen reported lacking information about how the municipality acted upon the care plan sent from the hospital. In some cases, they were unsure if the information had been read by the initial assessor and if the appropriate actions were put in place for the patient. They explained that this resulted in a feeling of uncertainty and unease when discharging the patient. Some nurses-initiated contact with the municipality themselves to find out what actions were put in place for the patient and when the municipality planned to visit the patient after discharge. They also felt that this information was appreciated by the patients and relatives, as they often did not know what to expect after discharge.

One respondent in Stockholm gave a similar example of making additional contact because of feelings of responsibility and not fully trusting the system:

*Today we had a lady who was very worried and was going home. We had had a meeting and she was going to need a lot of home help and then she was worried, and relatives were very worried. Then you start to think that it may not go well. She had some problems with her memory as well, and then it is difficult to know what to do. But then I tried to call the need assessor again and ask them to do a quick follow-up, that they do not let it take too long but check quite quickly how it works at home. And then I talked to the district nurse about the same thing as well, that it feels good if they can come as soon as possible and not wait too long. Then they decided they would have a follow-up immediately after the weekend, and you felt that they listened at least. The district nurse could not come tomorrow but then she would send their assistant nurses there tomorrow. Then you feel that at least you have tried.*

*(Hospital nurse SE, int1017).*

Other aspects of additional communication initiated by staff included providing very detailed information to the home help team who provide social care to make their work and the life of the patient as smooth as possible.

### Work beyond duty

All respondents provided examples of how they initiated responsibility when they encountered system errors or when they felt that following existing regulations/guidelines was not enough to meet the patients’ needs. Several participants gave examples of situations when they undertook work not necessarily part of their job due to strong feelings of responsibility for patients. This particularly related to patients with no close relatives and patients less capable of expressing and taking care of themselves.

*I say what I have to inform about and then I go out so that I have time to do other things. But those [patients without relatives] I usually do not leave, then I am with them during the whole meeting, even if it takes some time, because I feel that then you are still some kind of representative, even if you are not a relative. You are there to understand a little of what they [the municipality] are saying. Because there is a lot of information and they [the patients] may not really remember everything that has been said, but then there is two of us who have heard it.*

*(Hospital nurse SE, int1017).*

Undertaking work not part of their job also included facilitating provision of additional information to patients and by interpreting conversations between colleagues and patients to and from foreign languages.

## Discussion

The aim of this study was to examine and compare healthcare professionals’ perceptions on barriers and facilitators of good care coordination for older patients with complex health and social care needs being discharged from hospital in Copenhagen and Stockholm. Trough the qualitative analyses of the interview material we found three overarching themes related to the participants’ perception of barriers and facilitators: 1) intended communication ways, which was primarily perceived as facilitators for good care coordination when it worked well and as barriers when information was lacking or communication was delayed or difficult to access; 2) The organisational structures which could also be seen as both barriers and facilitators and 3) supplementary actions which was only viewed as facilitators and where put in place by the participants when they felt that the system failed.

### Intended communication ways

Related to intended communication ways, we found that participants in both Copenhagen and Stockholm considered the shared electronic communication systems as a facilitator for good coordination of the transition from hospital to home. Previous research has also demonstrated that clear communication between the involved care teams is essential to secure a smooth transition from the hospital to the home [4–7]. Lack of information in the discharge report sometimes interfered with the planning process in Copenhagen. From previous research we have seen that poor discharge planning from the hospitals tends to cause delays in communication between hospital-based healthcare professionals and health and social care providers in the community, negatively affecting the management of the patients [7–9, 34]. From previous Danish studies it has been shown that especially homecare nurses difficulties accessing relevant information complicates a safe transition from hospital to home for the patients [11, 12]. In both settings the participants sometimes had difficulties accessing collaborators, which was experienced as a barrier for a safe transition from hospital to home.

### Organisation of the system

In Copenhagen the primary care physician is thought of as the patient’s permanent care contact, however, the participants reported that they had difficulties accessing the primary care physician and that the physicians did not take responsibility for disseminating information to patients or to collaborators. We have not been able to find any studies on this in a Danish context, however, in a UK study researchers found failure in general practitioners’ compliance with actions stated in the discharge summary report in 46% of the case [35]. From previous studies poor communication with primary care physicians during hospital discharge have been found to threatens the transition of care during hospital discharge [36]. We have no interviews with primary care doctors in Denmark, but the participants in Copenhagen mentioned the primary care physicians’ reimbursement system, which to a high degree is dependent on fee-for-service, as one possible explanation. Further, work pressure from having too many patients listed were also mentioned as a possible explanation. In Stockholm, work pressure was reported as the main barrier for close collaboration with primary care, from both hospital nursing staff and primary care nurses.

In Stockholm, there were coordinators designated to manage the hospital discharge planning, which in previous research has shown to facilitate a good discharge process [18, 19]. This was not brought up as a facilitator, but this might be because all our participants from the hospital had this role and did not reflect upon them self as a facilitator for the discharge process.

In Copenhagen, there were also two job categories that crossed the organisations. The follow-home nurse was following the patient home after discharge and could have a closer inspection of the home in relation to what help was needed, and then the discharge coordinators from the municipalities sometimes came to the hospital to meet the patient in the hospital setting. Both professions were seen as an opportunity for increasing collaboration and minimising the risk of patients “falling between two stools”. Previous research on the.

### Supplementary actions

All interviews included examples of how the participants took initiative and responsibility on their own for example calling collaborators if they were uncertain about whether information had reached them or taking action if they thought that the patient was not properly taken care of. In many ways they worked as a quality assurance of the system, always looking at the patient’s best interest.

In Copenhagen, both the follow-home nurse and the home care nurses in the municipalities reported that they were often placed in situations where they had to help the patients with tasks not included in their job description. They reported doing this to make sure that the patient was safe and looked after. In situations where it was unclear who was responsible, they took responsibility until it could be handed over to the appropriate authority. This has been shown in previous studies on homecare nurses and covers up the imperfections of the system [12] making it more difficult to realise the pitfalls in the system.

### Strengths and limitations

The qualitative research design of this study implies that possibility for generalisation is limited, and more research is needed to investigate to what degree these experiences are shared within each healthcare system. Nevertheless, the results correspond to previous findings about what facilitate good coordination of care and collaboration between care providers, and we therefore find it plausible that the results can be generalised to other context with similar organisation of healthcare. The transferability has also been supported by using a maximum variation approach in the sample selection both when it comes to different organisations and different job positions within the organisations.

To ensure credibility [33] the interview guide was pilot tested in both settings and the interviews were conducted by two experienced qualitative researchers. The interviews were transcribed verbatim and field notes were consulted when needed through the analysis process. Two researchers coded a selection of the interviews in each country, whereof one read and coded a selection of both Danish and Swedish interviews. When agreement about codes and interpretations were accomplished in the group, one researcher coded the rest of the Danish interviews, and another researcher coded the rest of the Swedish interviews – always in close collaboration (investigator triangulation). Further, the themes and sub-themes were presented and discussed within a larger research group where the members read a selection of the interviews (peer debriefing). This group had members of different genders, age, professional background and experiences with the health and social care system to reduce researcher bias and contribute to a broader interpretation of the material.

The study is limited to nurses’ (and two assistant nurses working as care coordinator) perceptions of what takes place in the handover between hospital and homebased healthcare. We have not included other professional groups that are involved in the delivery of care for example primary care physicians, physiotherapists or occupational therapists, which could have provided information on further barriers and facilitators. Another limitation is that our study is limited to the capitals in each country. Barriers and facilitators may vary between large cities and sector crossings in the countryside. Nevertheless, a similar study from Denmark focusing on homecare nurses in both urban and rural areas found that the experienced barriers and facilitators in rural and urban areas were similar [12]. To further provide information regarding transferability [33] we have provided a thick description of the study context and participants in the two settings.

Implications for practice.

The findings stress the importance of accessing information and collaborators across organisations. In Copenhagen, where there are no shared patient records between hospital nurses and homecare nurses, homecare nurses are forced to track down hospital nurses through telephone to get the information they need. This is not the case in Stockholm where homecare nurses also have access to patient records.

The results from this study point to the importance of understanding the patients’ needs and home situation after a discharge to ensure a safe return. Follow-home nurses, safe return teams and need assessors meeting patients at the hospital before discharge was lifted as important facilitators.

Implications for research.

In Stockholm, the nurses assigned as permanent care contact, who should be the link between hospital care and home care, do not feel that they have the time to properly engage in the patient´s discharge process. This was experienced as a barrier for coordinating a safe return to home and future research should investigate the magnitude of this problem further to understand if the aim of the permanent care contact is achieved.

In both systems we found that nurses sometimes feel the need to take own initiative, besides their job responsibility, when they do not feel that the system work properly. It is important to investigate to what degree the healthcare system rely on individual initiatives to avoid adverse situations for the patients.

## Conclusion

There are lessons to be learned from both care systems. The electronic communication systems are in general seen as a facilitator for coordinating care. However, the written e-communication between hospitals and home healthcare is sometimes perceived as a one-way communication in Copenhagen. The chat function in the Swedish e-communication system gives the possibility to get in contact more easily with other actors and collaborators.

In Copenhagen there are more sector-overlapping work which is perceived to facilitate a safer transition from hospital to home. Municipal discharge coordinators come to the hospital and can have a more regular contact with the hospital staff. The function of the follow-home nurse, that follow the patient from the hospital to the home is also seen as a facilitator for safe return as the follow-home nurse sees the patient´s home upon revival and can immediately act if the patient´s needs are not met.

In both systems nurses sometimes feel the need to take own initiative, besides their job responsibility, to make sure that the patient is properly taken care of. This information is important to address as it can point to areas where the organisation might not work properly.

## Supplementary Information

**Additional file 1.**

## Data Availability

The interview data analysed in this study are not publicly available, as the respondents have been promised anonymity and sharing the interview data would compromise anonymity. De-identified data is available from the corresponding author on reasonable request.
