# Supplementary material for: Healthcare professionals’ perception of barriers and facilitators for care coordination of older adults with complex care needs being discharged from hospital: A qualitative comparative study of two Nordic capitals
Source: BMC Geriatr. 2023 Jan 19;23:32. doi: 10.1186/s12877-023-03754-z (PMC9854150; doi:10.1186/s12877-023-03754-z)
Supplement: Supplementary file 1 — Additional file 1. [file 12877_2023_3754_MOESM1_ESM.docx]

**Supplementary file**

**1) Interview guide: questions for nurses**

Briefly describe what your job involves including your work tasks related to the discharge process of older adults.

Please describe the process older adults with complex health needs and in great need of health and social care face when discharged from hospital.

-who is involved in such process?

-how do these people/units interact?

-what are the priorities when deciding to discharge an older person?

-when prioritising, who may stay/be discharged?

-do people sometimes get discharged too early? Why?

-what are the differences between those prioritised to stay versus sent home or to care homes? (Health status? Resources needed (incl. staff)? Age? Gender? Family influence/support? Patient’s living arrangements own home/care institution?)

-are there any differences in the process depending on a) ethnical background or citizenship of the patient, b) gender, c) socio-economic position? (why? Examples?)

-what is done to involve the patient/take the patient’s views into account in the decision making and provision of care?

-what is done to maximise a safe return home after discharge?

-do you follow a routine for this or do you treat every case differently? (to what extent do you do so?)

-is the discharge process targeted and adapted to certain groups of patients (e.g. older, certain needs)?

-besides the need for medical treatment, what influences the decision to discharge a patient? (financial incentives?)

-who does the follow up of the patient’s health once the patient has returned home? (when?)

-what does the collaboration between hospital, primary care and social care look like?

-how is contact between hospital, primary care and social care established and undertaken?

-what is done to keep hospital, primary care and social care connected?

-what is done to involve the patient in the decision making and provision of care?

-what is done to prevent readmission to hospital?

-who is responsible for preventing readmission?

-What could be improved?

Anything to add?

**2) Researchers’ characteristic and reflexivity:**

AL: Assistant Professor, MPH, Global Public Health, Karolinska Institutet, Female researcher

JA: Assistant Professor, MPH, Aging Research Center, Karolinska Institutet, Female researcher

NKJ: PostDoc, MPH, Department of Public Health, Copenhagen University, Female researcher

None of the researchers have a background in care sciences and the researchers were not familiar with the discharge process from a professional perspective before this study. Through reading of grey literature and legal documents the researchers got an understanding of the rules and regulations related to the discharge process in the two countries. Through interviews with colleagues and family members with experience as working as a nurse in both hospital and homecare setting the researchers tried to get an understanding of how the discharge process work in reality.

The researchers had very few assumptions regarding the nurses’ experience of the discharge process before the project started, however, we did expect some differences between the different municipalities in Stockholm. In Region Stockholm one of the municipalities (Norrtälje) are providing integrated care, as the Region and the municipality have gone together in providing both health and social care to the inhabitants. We expected a higher degree of collaboration and better communication in Norrtälje as the hospital and most of the home healthcare providers are in the same organisation. Further, Stockholm municipality have a lot of different private homecare providers and we expected the communication and collaboration to be experienced as more difficult for the providers located in Stockholm municipality due to the many different collaborators.

We did not see substantial differences in Norrtälje to support that the communication and collaboration was experienced as being much better in that area compared to other areas. However, we did see that there were some barriers experienced by hospital nurses communicating with nurses in Stockholm municipality that was not experienced elsewhere. In some cases, the patients were prompted to re-list themselves at different primary care clinics, before a discharge. We did not have any confirmation of this from the primary care side, however, they confirmed that the workload was sometimes too high for them to engage in the discharge process as much as they aught to.

None of the researchers had personal relationship with the participants.

**3) Setting**

The interviews were conducted at the participants workplace or close by the workplace in relation to a shift.

**4) Data collection instruments**

Data was audio recorded on two different audio recorders in Sweden and Denmark. Fields notes were written down after each interview.

**5) Level of participation**

All invited units, except one, agreed to participate in the study. The manager from the unit that declined, declined due to an already too heavy workload among the nurses in that unit.

**6) Research paradigm**

The study was performed from a post-positivistic point of view. We believe that we can gain knowledge about reality that is highly probable and that common themes and understandings of barriers and facilitators for collaboration and communication in the discharge process can be identified through these interviews. However, we acknowledge that we cannot reach full understanding of reality and that the understanding we get through these interviews can be falsified in the future through further investigation.
